# Link between hyperuricemia, renal dysfunction, and hypertension

**DOI:** 10.1111/jch.14389

**Published:** 2021-11-21

**Authors:** Eisuke Amiya

**Affiliations:** ^1^ Department of Cardiovascular Medicine Graduate School of Medicine University of Tokyo Bunkyo‐ku Tokyo Japan; ^2^ Department of Therapeutic Strategy for Heart Failure University of Tokyo Bunkyo‐ku Tokyo Japan

The abnormalities in uric acid concentration are common in a variety of clinical settings. Uric acid, a byproduct of purine metabolism, was influenced by several factors, such as high purine or protein diets, alcohol consumption, conditions with high cell turnover, drugs, renal dysfunction, or enzymatic defects in purine metabolism. Further, the development of hypertension,^1^ renal dysfunction,^2^ and cardiovascular events^3^ have all been linked to an increase in serum uric acid. In the mechanistic insight, hyperuricemia was discovered to enhance various pathologic conditions by regulating several signals, such as inflammatory response, oxidative stress, insulin resistance/diabetes, endoplasmic reticulum stress, and endothelial dysfunction.^4^ However, there had been long‐term controversies about the clinical significance of hyperuricemia. In addition, the clinical efficacy of lowering serum uric acid had been clearly established.

In this issue of the *Journal of Clinical Hypertension*, Kawazoe and coworkers examined the risk of new‐onset hypertension based on serum uric acid levels using a population‐based cohort over a relatively long follow‐up period, and the study demonstrated the association between hyperuricemia and new‐onset hypertension.^5^ Furthermore, high uric acid levels increased the risk of developing new‐onset hypertension in the presence of chronic kidney disease.

High uric acid levels have been linked to an increased risk of hypertension. According to a meta‐analysis of 18 prospective cohort studies, the incidence of hypertension increased by 13% per 1 mg/dl increase in serum uric acid level.^6^ Moreover, a long‐term follow‐up longitudinal study supports the finding.^7^

However, the association between high uric acid and hypertension differed between populations. Grayson and coworkers discovered that the association was stronger in younger population.^6^ On the other hand, estrogen may have a protective effect against high levels of uric acid, lowering the risk of hypertension in women.^8^ The result of the current study added a novel finding of a triad of high uric acid levels, renal dysfunction, and hypertension development.

Indeed, there had been some debate about the link between uric acid levels, renal dysfunction, and hypertension.^9^ One possible mechanism is a common pathologic pathway derived from uric acid, which leads to hypertension, and renal dysfunction. Indeed, uric acid has been identified as a mediator of endothelial dysfunction.^10^ Moreover, uric acid promotes the formation of intracellular superoxide while inhibiting the phosphorylation of endothelial NO synthase.^11^ There have also been reports of uric acid‐mediated renin‐angiotensin system (RAS) activation.^12^ Indeed, RAS activation is associated with both hypertension and renal dysfunction, which could explain the synergistic effects of uric acid and renal dysfunction on hypertension development. Recently, uric acid has been discovered to activate the immune system. For instance, uric acid is internalized by mononuclear phagocytes and binds to NOD‐like receptors, activating the NLRP3 inflammasome.^13^ Through vascular smooth muscle remodeling, this pathway could lead to hypertensive inflammation.^14^ In addition, this pathway also promotes chemokine signaling in renal proximal tubular cells, resulting in tubular injury.^15^

Of course, there is a chance that high uric acid will cause an increase in blood pressure due to renal damage. There were several basic research findings regarding the effect of high uric acid levels on renal hemodynamics. Hyperuricemia causes glomerular hypertension by affecting preglomerular vessels and afferent arterioles. These findings suggest a mechanism by which hyperuricemia may mediate hypertension and renal disease.^6^ High serum uric acid levels were associated with an increase in juxtaglomerular renin and a decrease in macula densa neuronal nitric oxide synthase as well as renal tubule‐interstitial injury, which could also lead to hypertension and renal disease.^16^ Indeed, there have been a number of clinical studies on the impact of hyperuricemia on the development of chronic kidney disease.^17^ Sedaghad and coworkers found a stronger link between hyperuricemia and decline in renal function in hypertensive individuals, indicating a synergistic effect from a different direction.^18^

The next critical question is whether lowering high uric acid slows the progression of hypertension or renal dysfunction. However, recent data do not support the efficacy of pharmacological lowering of serum uric acid.^19^, ^20^ We did not have a complete picture of the association between hyperuricemia and various clinical settings.

We have not uncovered enough evidence to determine the clinical significance of hyperuricemia. The association surrounding hyperuricemia should be warranted on a continuous basis. Kawazoe's report might add some findings about the association to elucidate the landscape of hyperuricemia.

## DISCLOSURE

EA is part of the Department, which is supported by NIPRO Corp; Terumo Corp.; Senko Medical Instrument Mfg.; Century Medical, Inc.; ONO Pharmaceutical Co., Ltd.; Medtronic Japan Co., Ltd; Nippon Shinyaku Co., Ltd; Mochida Pharmaceutical Co.; Boehringer Ingelheim Pharmaceuticals Inc.; Abiomed Inc.; AQuA Inc.; Fukuda Denshi Co., Ltd; and Sun Medical Technology Research Corp.
